# Inhibitory Effect of Lycopene on Amyloid-β-Induced Apoptosis in Neuronal Cells

**DOI:** 10.3390/nu9080883

**Published:** 2017-08-16

**Authors:** Sinwoo Hwang, Joo Weon Lim, Hyeyoung Kim

**Affiliations:** Department of Food and Nutrition, Brain Korea 21 PLUS Project, College of Human Ecology, Yonsei University, Seoul 03722, Korea; sinwoo920322@gmail.com (S.H.); jwlim11@yonsei.ac.kr (J.W.L.)

**Keywords:** amyloid-β, apoptosis, NF-κB, Nucling, lycopene, reactive oxygen species

## Abstract

Alzheimer′s disease (AD) is a fatal neurodegenerative disease. Brain amyloid-β deposition is a crucial feature of AD, causing neuronal cell death by inducing oxidative damage. Reactive oxygen species (ROS) activate NF-κB, which induces expression of Nucling. Nucling is a pro-apoptotic factor recruiting the apoptosome complex. Lycopene is an antioxidant protecting from oxidative stress-induced cell damage. We investigated whether lycopene inhibits amyloid-β-stimulated apoptosis through reducing ROS and inhibiting mitochondrial dysfunction and NF-κB-mediated Nucling expression in neuronal SH-SY5Y cells. We prepared cells transfected with siRNA for Nucling or nontargeting control siRNA to determine the role of Nucling in amyloid-β-induced apoptosis. The amyloid-β increased intracellular and mitochondrial ROS levels, apoptotic indices (p53, Bax/Bcl-2 ratio, caspase-3 cleavage), NF-kB activation and Nucling expression, while cell viability, mitochondrial membrane potential, and oxygen consumption rate decreased in SH-SY5Y cells. Lycopene inhibited these amyloid-β-induced alterations. However, amyloid-β did not induce apoptosis, determined by cell viability and apoptotic indices (p53, Bax/Bcl-2 ratio, caspase-3 cleavage), in the cells transfected with siRNA for Nucling. Lycopene inhibited apoptosis by reducing ROS, and by inhibiting mitochondrial dysfunction and NF-κB-target gene Nucling expression in neuronal cells. Lycopene may be beneficial for preventing oxidative stress-mediated neuronal death in patients with neurodegeneration.

## 1. Introduction

Alzheimer’s disease (AD) is a major neurodegenerative disorder, which is characterized by a progressive cognitive decline, leading to dementia. In the pathogenesis, amyloid-β oligomers tend to accumulate and are associated with neuronal degeneration. In the onset and progress of neurodegenerative process in AD, neuroinflammation and oxidative stress occur in the neural tissue. In early phase of AD, little is known about the interplay of microglia, astrocytes and neurons in response to amyloid-β. In late phase of AD, glial cells produce large amounts of inflammatory cytokines, reactive oxygen and nitrogen species, which disrupts nerve terminals activity causing memory decline and neuronal death [1]. 

AD is the sixth leading cause of death in Americans and the fifth leading cause of death in the United States age ≥65 years. In 2013, Alzheimer’s Association reported that 5.2 million Americans have AD. Approximately 200,000 patients are aged <65 years (early-onset AD) while 5 million people comprises later-onset AD [2]. The association reported that an American develops AD every 68 s. For those age ≥85 years, prevalence of AD increases dramatically across all racial and ethnic groups. The population and the number of AD patients are growing rapidly in most Asian countries. Regarding early-onset AD, the mutation of presenilin-1 protein (PSEN)1, and PSEN2 were detected in the AD patients of Korea and the People’s Republic of China [3]. The transmembrane portion 1 (TMD-1) of PSEN is a region important for the γ-secreatase cleavage of amyloid-β. Recently, Lui et al. [4] reported that changes in a single amino acid in codons 88 and 89 of TMD-1 can result in dramatic increase in amyloid-β, which may be associated with early onset of AD. 

Even though it is not established whether amyloid-β induces onset of AD, evidence has shown that amyloid-β plays the main role in neuronal dysfunction and AD [5,6]. Amyloid-β is a biological molecule that interacts with several different receptor proteins and/or forms insoluble assemblies [7]. Therefore, its nonphysiological deposition may change normal neuronal conditions, which causes neuronal dysfunction in AD. 

For the therapeutic agents of AD, only five symptomatic drugs have been approved. Four of them are acetylcholinesterase inhibitors and one is an *N*-methyl-d-aspartate (NMDA) receptor antagonist. Since these drugs are symptomatic treatments, temporarily ameliorating memory and thinking problems, and their clinical effect is modest. Because they act solely on symptoms, without having any profound disease-modifying effects, they do not treat the underlying cause of AD and do not slow the rate of cognitive decline [8]. Therefore, drug discovery and development efforts has shifted toward disease-modifying therapy for AD. Aim of treatments is to affect the underlying disease process by impacting one or more of the many brain changes shown in AD. In particular, many researchers has been focused on phytochemical compounds having antioxidative, anti-amyloidogenic, anti-inflammatory and anti-apoptotic properties [9,10]. Recent study showed benefits and risks of plant food supplements with antioxidant properties for treatment of neurodegenerative diseases. Piemontese [11] reported the contamination of mycotoxin, heavy metals and pesticides in plant food supplement in the marketed products and suggested the need for strict European regulations for the quality and benefits of these kinds of food supplements. 

Although the underlying molecular mechanism is still unclear, recent studies have shown that reactive oxygen species (ROS) and neuronal degradation by mitochondrial dysfunction are largely responsible for the process of AD [12]. Oxidative stress is the imbalance between cellular ROS production and antioxidant capacity. It is related to neurodegenerative diseases because ROS oxidizes major cellular components, lipids, nucleic acids, and proteins, which cause mitochondrial dysfunction and necrotic or apoptotic cell death [13,14]. 

In the central nervous system, mitochondria are major sources of ROS. Superoxide is produced by reaction of single electrons and oxygen via redox carriers. Under normal conditions, ROS production and removal are balanced; however, the balance is greatly collapsed in AD, causing ROS deposition and oxidative damage. Mitochondria also play a pivotal role in the intrinsic pathway of apoptosis [15]. During apoptosis, the mitochondrial system is disrupted and the outer membrane is permeabilized, leading to release of apoptotic proteins [16]. Increased mitochondrial membrane permeability, especially by amyloid-β, accelerates the opening of the mitochondrial permeability transition pore (MPTP) and may lead to apoptosis [17]. 

The transcription factor NF-κB belongs to the NF-κB/Rel family, which is a dimeric protein. In resting cells, NF-κB is insulated against the cytoplasm by an inhibitory IκBα protein. The IκB kinase complex (IKK) is activated under the stimulus, leading to proteasomal degradation of phosphorylated IκBα and translocation of NF-κB to the nucleus [18]. In general, NF-κB is considered to be an anti-apoptotic factor. However, it triggers apoptosis in a stimulus-dependent manner [19]. Nucling, a pro-apoptotic factor, promotes stress-induced apoptosis by recruiting the Apaf1/procaspase 9 complex [20]. A recent study showed that expression of Nucling is mediated by NF-κB [21]. 

Lycopene is a carotenoid pigment, mostly found in fruits and vegetables with red color. Lycopene is a powerful antioxidant because of its conjugated double bonds. By its antioxidant activity, lycopene protects lipoproteins and vascular cells from oxidation. Lycopene is the effective singlet oxygen quencher in plasma, low-density lipoprotein, and human lymphoid cells in vitro [22,23,24]. Consumption of lycopene-rich foods has shown to decrease the risk of cardiovascular diseases and cancers [25,26,27]. Chen et al. [28] reported that lycopene supplementation significantly decreases the DNA tail length, with a mean difference of −6.27 (95% confidence interval −10.74, −1.90) (*p* = 0.006) between the lycopene intervention groups and the control groups. They reported that lycopene possibly alleviates oxidative stress in lycopene intervention trials for disease prevention.

Since lycopene is lipophilic, eating with fat increases uptake rather than without oil preparations in the diet [29]. Lycopene is mainly stored in the liver, lungs, prostate gland, colon and skin in the human body and concentration of lycopene in body tissues is higher than those of other carotenoids [30]. Moreover, lycopene can pass through the BBB, due to its lipophilic nature, suggesting that it may prevent oxidative stress-induced neurologic lesions [31,32]. Hsiao et al. showed that lycopene had protective effect on focal cerebral ischemia in rat models [33]. Qu et al. indicated that lycopene inhibited amyloid-β-induced neurotoxicity and mitochondrial dysfunction in cultured rat cortical neurons [34]. Lycopene has been shown to have neuroprotective effects in 3-nitropropionic acid-induced Huntington’s disease-like symptoms in rats [35] and 1-methyl-4-phenyl-1,2,3,6-tetrahydropyridine (MPTP)-induced mouse model of Parkinson’s disease [36]. In both studies, lycopene inhibited oxidative stress and impairment of mitochondrial enzyme activities. Lycopene reversed neurochemical changes and physiological abnormalities in experimental animals.

The aim of this study was to investigate whether lycopene inhibits amyloid-β-stimulated apoptotic cell death by reducing intracellular and mitochondrial ROS and by suppressing mitochondrial dysfunction (decrease in mitochondrial membrane potential and oxygen consumption rate), NF-κB-mediated Nucling expression, and apoptotic indices (increase in p53, Bax/Bcl-2 ratio, and caspase-3 cleavage) in human neuronal SH-SY5Y cells. To determine the role of Nucling in amyloid-β-induced apoptosis, the cells, which were transfected with non-targeting control siRNA (NT siRNA) of siRNA for Nucling (Nucling siRNA), were prepared and cell viability and apoptotic indices were monitored.

## 2. Materials and Methods 

### 2.1. Reagents

Lycopene and amyloid-β were purchased from Sigma-Aldrich (St. Louis, MO, USA). Lycopene was dissolved in tetrahydrofuran (Sigma-Aldrich, St. Louis, MO, USA) (final concentration 5 mM), and amyloid-β was dissolved in water (final concentration 1 mM), incubated at 37 °C in a humidified atmosphere of 95% air and 5% CO_2_ for 72 h, and stored at −20 °C.

### 2.2. Cell Line and Culture Condition

Human neuroblastoma SH-SY5Y cells were cultured in Dulbecco’s Modified Eagle’s medium (Sigma-Aldrich, St. Louis, MO, USA) supplemented with 10% fetal bovine serum (GIBCO, Grand Island, NY, USA) and antibiotics (100 U/mL penicillin and 100 μg/mL streptomycin). The cells were cultured at 37 °C in a humidified atmosphere of 95% air and 5% CO_2_.

### 2.3. Small Interfering RNA (siRNA) Targeting Nucling 

Nucling siRNA (ab169684) and NT siRNA were obtained from Abcam (Cambridge, MA, USA). NT siRNA was used as an untargeted control. The cells were seeded into 6-well plates and cultured for 24 h. At 70–80% confluence, the cells were transfected with Nucling siRNA or NT siRNA by using DOTAP (*N*-[1-(2,3-dioleoyloxy) propyl]-*N*,*N*,*N* trimethyl ammonium methylsulfate) (Boehringer-Mannheim, Pentzberg, Germany) and cultured for 48 h. Transfection efficiency was confirmed by western blot analysis for Nucling in the transfected cells, with or without amyloid-β stimulation.

### 2.4. Experimental Protocol

To investigate the effect of lycopene, the cells (1 × 10^5^/mL), either wild-type or transfected, were pretreated with lycopene (0.2 or 0.5 μM) for 1 h, then stimulated with amyloid-β (20 μM) for another 24 h.

### 2.5. Measurement of Cell Viability

The cells were seeded in 24-well culture plates at 4 × 10^4^ cells per well and cultured for 16 h. The cells were treated with amyloid-β for 24 h or 48 h. Viable cell number were determined by Trypan Blue exclusion test (0.2% Trypan Blue; Sigma-Aldrich).

### 2.6. Preparation of Cell Extracts

The cells were harvested by scraping with phosphate buffered saline (PBS), and pelleted by centrifugation at 5000× *g* for 15 min. The cell pellets were resuspended with lysis buffer containing 10 mM Tris (pH 7.4), 1% NP-40 and commercial protease inhibitor complex (Complete; Roche, Mannheim, Germany), and lysed by drawing the cells through a 1-mL syringe with several rapid strokes. The mixture was then incubated on ice for 30 min and centrifuged at 13,000× *g* for 15 min. The supernatants were collected and used as whole cell extracts. To prepare the cytoplasmic and nuclear extracts, the cell pellets were resuspended with 30 μL of hypotonic buffer containing 10 mM 4-(2-hydroxyethyl)-1-piperazineethanesulfonic acid (HEPES) pH 7.9, 1.5 mM MgCl_2_, 10 mM KCl, 0.5 mM DTT, 0.5 mM PMSF, 0.2% NP-40, then, placed on ice for 20 min. The extracts were centrifuged at 13,000× *g* for 20 min at 4 °C. The pellets were washed once with hypotonic buffer, resuspended in 30 μL of extraction buffer containing 20 mM HEPES pH 7.9, 420 mM NaCl, 0.2 mM EDTA, 1.5 mM MgCl_2_, 25% glycerol, 0.5 mM DTT, 0.5 mM PMSF and placed on ice for 20 min. The extracts were subsequently centrifuged at 13,000× *g* for 20 min at 4 °C, and the supernatants were used as the nuclear extracts. The protein concentration was determined by the Bradford assay (Bio-Rad Laboratories, Hercules, CA, USA).

### 2.7. Measurement of Intracellular ROS and Mitochondrial ROS Levels

For the measurement of intracellular ROS, the cells were loaded with 10 µg/mL of dichlorodihydrofluorescein diacetate (DCF-DA; Sigma-Aldrich) and incubated in 5% CO_2_/95% air at 37 °C for 30 min. DCF fluorescence was measured using a VICTOR 5 Multilabel Plate Reader (PerkinElmer Life and Analytical Sciences, Boston, MA, USA) at excitation of 495 nm and emission of 535 nm. Intracellular ROS level was expressed as the relative increase. 

For the measurement of mitochondrial ROS, the cells were loaded with 10 µM MitoSOX (Life Technologies, Grand Island, NY, USA) and incubated in 5% CO_2_/95% air in 37 °C for 30 min. The fluorescent mitoSOX was measured using a VICTOR 5 Multilabel Plate Reader (PerkinElmer Life and Analytical Sciences) at excitation of 514 nm and emission of 585 nm.

### 2.8. Western Blot Analysis for Bax, Bcl-2, caspase-3, p53, IκBα, p-IκBα, NF-κB, and Nucling

Whole cell extracts (20–40 µg) were loaded per lane, separated by 8–12% SDS-polyacrylamide gel electrophoresis under reducing conditions, and transferred onto nitrocellulose membranes (Amersham, Inc., Arlington Heights, IL, USA) by electroblotting. The transfer of protein was verified using reversible staining with Ponceau S. Membranes were blocked using 2% nonfat dry milk in TBS-T (Tris-buffered saline and 0.2% Tween 20) for 1 h at room temperature. The proteins were detected with the antibodies for Bax (sc-526, Santa Cruz Biotechnology, Dallas, TX, USA), Bcl-2 (sc-492, Santa Cruz Biotechnology), caspase-3 (#9662S, Cell Signaling Technology, Danvers, MA, USA), IκBα (sc-371, Santa Cruz Biotechnology), p-IκBα (#2859, Cell Signaling Technology), p53 (sc-1311, Santa Cruz Biotechnology), Nucling (sc-135510, Santa Cruz Biotechnology), and actin (sc-1615, Santa Cruz Biotechnology) dilution in TBS-T containing 2% nonfat dry milk, and incubated overnight at 4 °C, followed by secondary antibodies (anti-goat, anti-mouse, or anti-rabbit conjugated to horseradish peroxidase). The protein bands were visualized using the ECL detection system (Santa Cruz Biotechnology), according to the manufacturer’s instruction. For quantification of protein, the protein band of each protein was scanned using Bio-Rad scanner (GS-700, Bio-Rad Laboratories, Hercules, CA, USA) driven by volume analysis software and quantified with Molecular Analysis software (version 4.1) (Perkin-Elmer, Waltham, MA, USA). Protein levels of total and phospho-specific forms of IκBα and Nucling were compared with that of the loading control actin and expressed as percentage ratios of the band densities. The ratio of Bax/Bcl-2 was determined by the protein-band densities of Bax and Bcl-2. Values are expressed as the mean ± S.E. of three independent experiments.

### 2.9. Electrophoretic Mobility Shift Assay (EMSA)

Nuclear extracts (0.3 µg) of the cells treated with lycopene and amyloid-β were incubated with the ^32^P-labeled double-stranded oligonucleotide 5′-GGGCCAAGAATCTTAGCAGTTTCGGG-3 in buffer containing 12% glycerol, 12 mM HEPES (pH 7.9), 1 mM EDTA, 1 mM DTT, 25 mM KCL, 5 mM MgCl_2_, and 0.04 µg/mL poly[d(I-C)] at 15–25 °C for 30 min. The extracts were then subjected to electrophoretic separation at room temperature on a nondenaturing 5% acrylamide gel at 30 mA using 0.5 × Tris borate/EDTA buffer. The gels were dried at 80 °C for 1 h and exposed to radiography film for 24 h at −70 °C with intensifying screens. Intensity of NF-κB band was densitometrically quantified relative to None group. Intensity of NF-κB band of None group was set as 1. Values are expressed as the mean ± S.E. of three independent experiments. 

### 2.10. Measurement of Mitochondrial Membrane Potential (MMP)

The membrane-permeant dye 5,5′,6,6′-tetrachloro-1,1′,3,3′-tetraethyl benzimidazolyl carbocyanine iodide (JC-1) is widely used in apoptosis studies to monitor mitochondrial health. JC-1 dye exhibits potential-dependent accumulation in mitochondria, indicated by a fluorescence emission shift from green (~529 nm) to red (~590 nm). Consequently, mitochondrial depolarization is indicated by a decrease in the red/green fluorescence intensity ratio. The potential-sensitive color shift is due to concentration-dependent formation of red fluorescent J-aggregates.

To determine changes in MMP, the cells were cultured on glass coverslips coated with Poly-l-lysine, pretreated with lycopene for 1 h, and stimulated with amyloid-β (20 µM) for another 24 h. Then, the cells were incubated with JC-1 reagent (1:100; 10009908, Cayman Chemical Company, Ann Arbor, MI, USA) for 20 min. After removing the media, the cells were dried for 15 min at room temperature and washed with PBS for 5 min, twice. The cells were mounted with mounting solution (M-7534, Sigma Aldrich). JC-1 fluorescent (red; excitation at 590 nm and emission at 610 nm, green; excitation at 485 nm and emission at 535 nm) was examined with a laser-scanning confocal microscope (LSM 880, Carl Zeiss Inc., Oberkochen, Germany). Fluorescence images were expressed as the percentage ratio of the fluorescence densities of red and green using NIH Image J 5.0 software (National Institutes of Health, Bethesda, MD, USA). Average intensity per cell was determined and more than 50 cells in each experimental group were analyzed.

### 2.11. Measurement of Oxygen Consumption Rate (OCR)

OCR, an index of mitochondrial respiration, was determined using XF96 extracellular Flex Analyzer (Seahorse Bioscience Inc., Billerica, MA, USA). Cells were washed with base media once, immersed in 175 μL base media, and incubated in the absence of CO_2_ for 20 min. The cartridge was loaded to dispense three metabolic inhibitors, sequentially, at specific time points: oligomycin (inhibitor of ATP synthase, 1 μM), which blocks ATP synthase and shows the OCR dedicated to ATP production; followed by FCCP (a protonophore and uncoupler of mitochondrial oxidative phosphorylation, 0.5 μM), which permeabilizes the inner mitochondrial membrane and shows maximal OCR; followed by the addition of a combination of rotenone (mitochondrial complex I inhibitor, 100 nM) and antimycin A (mitochondrial complex III inhibitor, 100 nM) for OCR measurement using the XF Cell Mito Stress Test Kit (Cat. No. 103015-100, Seahorse Bioscience Inc., Billerica, MA, USA). Measurements of OCR were normalized to the cell number and protein concentration for the cells. OCA was expressed as the unit of pMoles/min.

### 2.12. Statistical Analysis

One-way ANOVA, followed by Newman–Keuls′ post hoc tests, was used for statistical analysis. All values in the results were expressed as the mean ± S.E. of three different experiments. A *p*-value of 0.05 or less was considered statistically significant.

## 3. Results

### 3.1. Lycopene Inhibits Amyloid-β-Induced Cell Death and Increase in Apoptotic Indices (p53, Bax/Bcl-2 Ratio, Caspase-3 Cleavage) in SH-SY5Y Cells 

**Figure 1:**
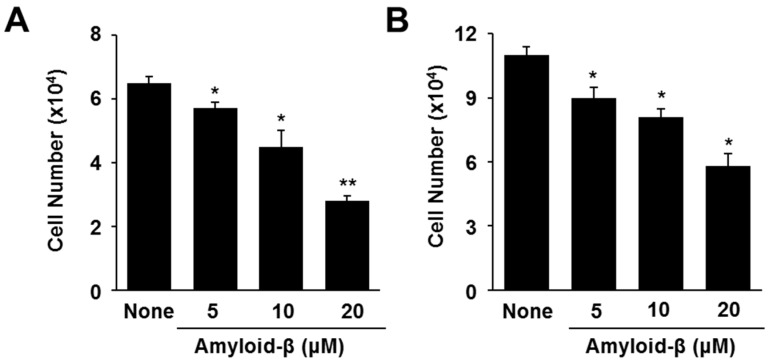
Cell viability in amyloid-β–stimulated cells. The cells were stimulated with the indicated concentration of amyloid-β for 24 h (**A**) or 48 h (**B**). Viable cell numbers was determined by the Trypan Blue exclusion test. Data are expressed as the mean ± S.E. of three independent experiments. * *p* < 0.05, ** *p* < 0.01 vs. None (without amyloid-β stimulation).

In order to investigate whether amyloid-β induces cell death, various concentrations of amyloid-β and 24 h-culture (A) and 48 h-culture (B) periods were used (Figure 1). At both the 24 h- and 48 h-cultures, amyloid-β decreased viable cell numbers concentration dependently. The 20 μM of amyloid-β showed maximal decrease in cell viability at the 24 h-culture. Therefore, the 24 h-period and 20 μM concentration of amyloid-β were used for further experiments. 

**Figure 2:**
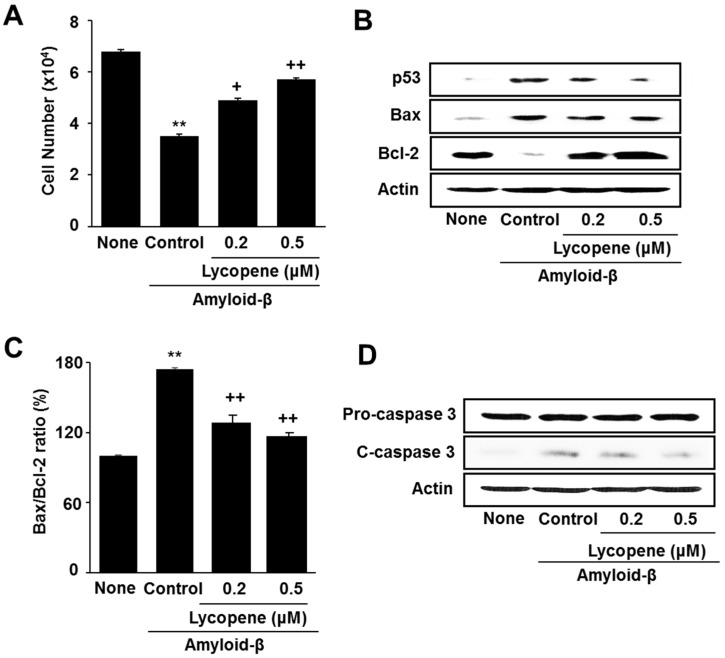
Effect of lycopene on cell viability and apoptotic indices in amyloid-β-stimulated cells. The cells were pretreated with lycopene for 1 h and then stimulated with amyloid-β (20 μM) for another 24 h. (**A**) Viable cell numbers was determined by the Trypan Blue exclusion test; (**B**) Levels of p53, Bax, Bcl-2, caspase-3, and actin were determined by western blot analysis; (**C**) The ratio of Bax/Bcl-2 was determined by protein band densities of Bax and Bcl-2; (**D**) Levels of pro- and cleaved-caspase-3 were assessed by western blot analysis. Actin was used as a loading control. The value of None (without any stimulation or treatment) was set as 100%. Data are expressed as the mean ± S.E. of three independent experiments. ** *p* < 0.01 vs. None (without any stimulation or treatment); ^+^ *p* < 0.05, ^++^ *p* < 0.01 vs. Control (with amyloid-β stimulation alone). c-caspase-3, cleaved caspase-3.

Lycopene inhibited amyloid-β-induced cell death in a dose-dependent manner (Figure 2A). Stimulation of amyloid-β increased pro-apoptotic Bax and decreased anti-apoptotic Bcl-2 in SH-SY5Y cells. Amyloid-β also increased the level of p53, a pro-apoptotic gene that induces cell cycle arrest, in the cells (Figure 2B). Protein band intensity for Bax/Bcl-2 of the None group was set as 100%. Amyloid-β-induced increase in Bax/Bcl-2 was reduce by lycopene treatment (Figure 2C). 

Caspase-3 exists as an inactive proenzyme, as pro-caspase-3. Upon exposure to apoptosis-inducing stimuli, pro-caspase-3 undergoes proteolytic processing to form the active enzyme. Therefore, the level of cleaved caspase-3 reflects the activation of caspase-3. The level of cleaved caspase-3 increase by amyloid-β, which was inhibited by lycopene treatment in the cells (Figure 2D).

### 3.2. Lycopene Reduces ROS Levels and Inhibits Mitochondrial Dysfunction in Amyloid-β-Stimulated SH-SY5Y Cells

**Figure 3:**
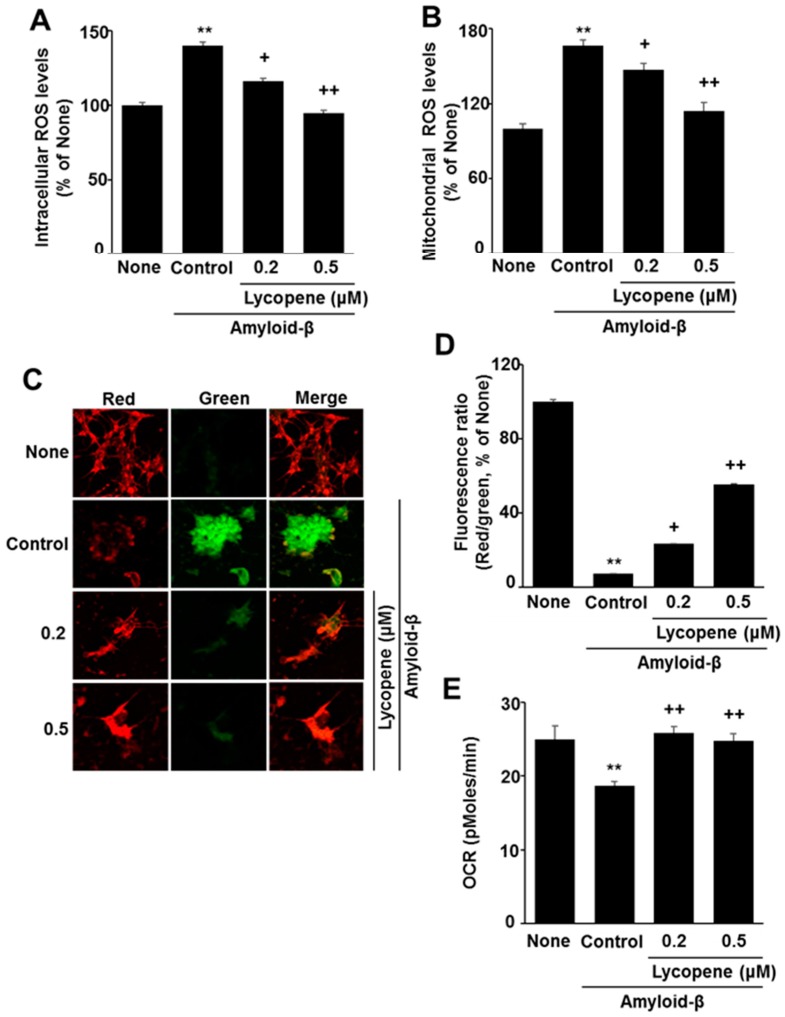
Effect of lycopene on intracellular and mitochondrial reactive oxygen species (ROS) levels, mitochondrial membrane potential (MMP), and oxygen consumption rate (OCR) in amyloid-β-stimulated cells. The cells were pre-treated with lycopene for 1 h, and then stimulated with amyloid-β (20 μM) for another 24 h. (**A**) Intracellular ROS levels were determined by dichlorodihydrofluorescein diacetate assay; (**B**) Mitochondrial ROS levels were measured by mitoSOX assay; (**C**,**D**) MMP was determined by JC-1 dye through confocal microscopy. (**E**) OCR was measured by using an XF analyzer. The value of the None group (without stimulation) was set as 100% (**A**,**B**,**D**). Data are expressed as the mean ± S.E. of three independent experiments. ** *p* < 0.01 vs. None (without any stimulation or treatment); ^+^ *p* < 0.05, ^++^ *p* < 0.01 vs. Control (with amyloid-β stimulation alone).

To determine the levels of intracellular and mitochondrial ROS, DCF-DA and mitoSOX assays were used. Amyloid-β stimulation increased both intracellular and mitochondrial ROS levels (Figure 3A,B). Lycopene significantly decreased both levels of intracellular and mitochondrial ROS in a dose-dependent manner to determine whether amyloid-β affects mitochondrial function, MMP and OCR were assessed by confocal microscopy and the XF analyzer, respectively. As shown in Figure 3C,D, lycopene significantly inhibited a decrease in MMP in amyloid-β-stimulated SH-SY5Y cells. The JC-1 dye was used as an indicator of mitochondrial potential, as the ratio of green to red fluorescence depends on the membrane potential. The potential-sensitive color shift is due to the formation of red fluorescent J-aggregates. As shown in Figure 3C, amyloid-β increased the ratio of green to red fluorescence in the cells, which reflects a decrease in MMP in amyloid β-stimulated cells (second panel). Lycopene prevented amyloid β-stimulated decrease in MMP in the cells, in a dose-dependent manner (third and fourth panels). Fluorescence images are expressed as the percentage ratio of the fluorescence densities of red and green (Figure 3D). The fluorescence ratio of red/green was lower in amyloid-β-stimulated cells than in the None group (without stimulation). Lycopene prevented amyloid-β-induced decrease in the ratio of red/green, dose-dependently. 

Mitochondrial respiration, determined by OCR, decreased by amyloid-β in the cells (Figure 3E). Lycopene suppressed decrease in OCR in amyloid-β-stimulated cell, suggesting that lycopene prevents mitochondrial damage induced by amyloid-β in the cells. 

### 3.3. Lycopene Inhibits Phosphorylation of IκBα, NF-kB Activation, and Nucling Induction in Amyloid-β-Stimulated SH-SY5Y Cells

**Figure 4:**
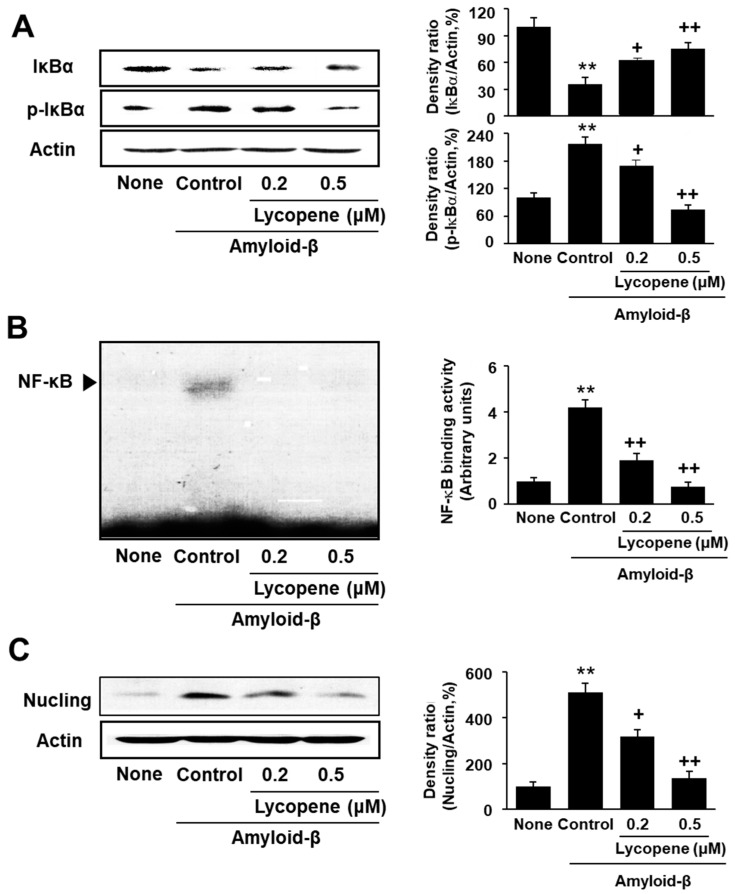
Effect of lycopene on total and phospho-specific form of IκBα, NF-kB-DNA binding activity, and Nucling expression in amyloid-β-stimulated cells. The cells were pretreated with lycopene for 1 h, and then stimulated with amyloid-β (20 μM) for another 24 h. (**A**) The levels of total and phospho-specific forms of IκBα were determined by western blot analysis. Protein levels of total and phospho-specific forms of IκBα were compared with that of the loading control actin and expressed as percentage ratios of the band densities; (**B**) NF-κB–DNA binding activity was determined by electrophoretic mobility shift assay. Intensity of NF-κB band was densitometrically quantified relative to None group. Intensity of NF-κB band of None group was set as 1; (**C**) The levels of Nucling were determined by western blot analysis. Protein levels of Nucling were compared with that of the loading control actin and expressed as percentage ratios of the band densities. Data are expressed as the mean ± S.E. of three independent experiments. ** *p* < 0.01 vs. None (without stimulation); ^+^ *p* < 0.05, ^++^ *p* < 0.01 vs. Control (with amyloid-β stimulation alone).

NF-κB is bound with an inhibitory IκBα in the resting state. Upon exposure to various stimuli, IκBα is phosphorylated by IKK for proteasomal degradation. Consequently, NF-κB is translocated to the nucleus and transcribes target genes, such as Nucling, in the cells. As shown in Figure 4A, amyloid-β increased the level of phospho-specific IκBα, but decreased the total IκBα level. These results may be related to increase in NF-kB-DNA binding activity by amyloid-β stimulation in the cells (Figure 4B). Similarly, the expression of Nucling, a mediator of apoptosis by recruiting apoptosome complex, was induced in amyloid-β-stimulated SH-SY5Y cells, which was suppressed by lycopene treatment (Figure 4C). Taken together, lycopene-inhibited amyloid-β-induced phosphorylation and degradation of IκBα, activation of NF-κB, and expression of Nucling in the cells (Figure 4A–C).

### 3.4. Transfection of Nucling siRNA Inhibits Cell Death and Increase in Apoptotic Indices in Amyloid-β-Stimulated SH-SY5Y Cells

**Figure 5:**
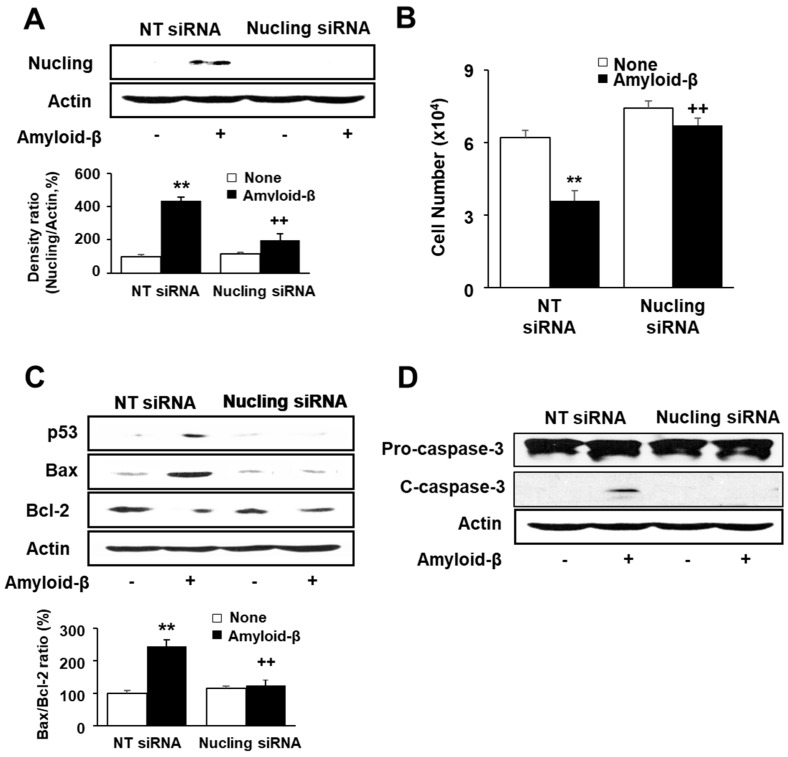
Cell viability and apoptotic indices in NT siRNA-transfected cells and Nucling siRNA-transfected cells with or without amyloid-β stimulation. The transfected cells were pre-treated with lycopene for 1 h and, then, stimulated with amyloid-β (20 μM) for another 24 h. (**A**) Levels of Nucling were determined by western blot analysis. Protein levels of Nucling were compared with that of the loading control actin and expressed as percentage ratios of the band densities; (**B**) Viable cell number was determined by the Trypan Blue exclusion test. The value for NT siRNA-transfected cells without amyloid-β stimulation was set as 100%; (**C**) Levels of p53, Bax and Bcl-2 were determined by western blot analysis. The ratio of Bax/Bcl-2 was determined by protein band densities of Bax and Bcl-2; (**D**) The levels of pro- and cleaved-caspase-3 were determined by western blot analysis. Actin served as a loading control. Values are expressed as the mean ± S.E. of three independent experiments. ** *p* < 0.01 vs. NT siRNA-transfected cells without amyloid-β stimulation; ^++^ *p* < 0.01 vs. NT siRNA-transfected cells with amyloid-β stimulation. NT siRNA, non-targeting control siRNA; c-caspase-3, cleaved caspase-3.

To investigate the role of Nucling on amyloid-β-induced apoptosis, the cells were transfected with NT siRNA or Nucling siRNA and stimulated with or without amyloid-β. Cell viability and indices of apoptosis (p53, Bax, Bcl-2, caspase-3 cleavage) were determined in the transfected cells. At first, the protein level of Nucling was assessed of the transfected cells to determine the transfection efficiency. As shown in Figure 5A, amyloid-β induced expression of Nucling in NT siRNA-transfected cells. However, amyloid-β did not affect the Nucling level in Nucling siRNA-transfected cells. The results show that transfection was efficiently performed. Figure 5B shows that in NT siRNA-treated cells, amyloid-β decreased cell viability. However, in Nucling siRNA-transfected cells, viable cell numbers were not affected by amyloid-β stimulation. Amyloid-β-induced cell death was not shown by Nucling siRNA transfection. Amyloid-β induced increase in p53 and Bax, but decrease Bcl-2 levels in NT siRNA-transfected cells (Figure 5C). Amyloid-β-induced alterations in apoptotic indices were inhibited in Nucling siRNA-transfected cells. Caspase-3 activation, which was determined by increase in cleaved caspase-3, was shown in NT siRNA-transfected cells by amyloid-β stimulation (Figure 5D). However, amyloid-β did not induce caspase-3 activation in Nucling siRNA-transfected cells.

## 4. Discussion

Amyloid-β induces its neurotoxicity in a variety of ways, including accelerating mitochondrial dysfunction, ROS production, and neuronal death [37]. In numerous studies, lycopene is regarded as a powerful antioxidant that passes through the BBB by its lipophilic nature. Several studies have shown that lycopene protects from some neurodegenerative diseases related to oxidative damage [32,38]. In the present study, lycopene significantly inhibited amyloid-β-induced increase in intracellular and mitochondrial ROS levels and apoptotic cell death in human neuronal SH-SY5Y cells. 

Present study showed that amyloid-β (at concentration of 20 μM) induced maximal decrease in cell viability at the 24 h-culture rather than 48 h-culture. It is not clear the reason why amyloid-β-induced cell death is independent on culture time. Recently, Zhang et al. [39] also demonstrated that amyloid-β (20 μM)-induced cell death was higher at 24 h-culture than at 48 h-culture, which supports the present results. Further study should be performed to determine the mechanism of cell death by amyloid-β in neuronal cells using the different culture times.

Apoptosis results from a cascade of events driven by several apoptosis-regulated genes. Among these genes, capsase and members of the Bcl-2 family are considered to be the most effective apoptotic regulators [40]. Bcl-2 protein, which is located in the mitochondrial outer membrane, is known to promote cell survival and protect against cell death caused by various apoptotic stimuli [41]. To determine whether amyloid-β-induced cell death is apoptotic, several apoptosis-regulated genes, such as Bcl-2 family genes and caspase-3, were determined. In the present study, stimulation of amyloid-β increased apoptotic indices including increase in the Bax/Bcl-2 ratio, caspase-3 activation, and p53 expression. Treatment with lycopene reduced all these apoptotic responses in amyloid-β-stimulated cells. These results indicate that amyloid-β caused neuronal apoptosis, which was inhibited by lycopene.

We hypothesized that lycopene may suppress neuronal apoptosis via its antioxidant capacity. However, the molecular mechanisms underlying the antioxidant effect of lycopene and its inhibition of neuronal apoptosis have not yet been clarified. Regarding the ability of lycopene to suppress oxidative stress, we found that lycopene inhibited an amyloid-β-induced increase in both intracellular and mitochondrial ROS. Several studies have shown that oxidative stress is related to neurodegenerative disorders, as ROS oxidize vital cellular components of neuronal cells [9]. Especially, increase in mitochondrial ROS is an important index of mitochondrial dysfunction in neuronal diseases [42,43]. Recently, we showed that lycopene inhibits apoptosis by reducing oxidative stress and downregulating Nucling in neuronal cells transfected with regulator of calcineurin [44]. In the present study, lycopene significantly inhibited amyloid-β-induced mitochondrial dysfunction, which was proven by its protective effect in reducing both MMP and OCR. The results strongly suggest that lycopene prevents amyloid-β-induced mitochondrial dysfunction and reduces ROS levels in neuronal cells. 

ROS have a potential role of activating NF-κB in various cells. Although NF-κB is mainly considered as an anti-apoptotic molecule, recent studies have demonstrated that NF-κB accelerates apoptosis in a stimulus-dependent way. NF-κB target gene Nucling mediates apoptosis, which was confirmed in the present amyloid-β-stimulated cells. In addition, Nucling siRNA transfection prevented amyloid-β-induced apoptosis, determined by cell viability and apoptotic indices (increase in p53 and Bax, decrease in Bcl-2, caspase-3 activation) in SH-SY5Y cells. These results suggest that lycopene inhibits amyloid-β-induced apoptosis of neuronal cells through reducing ROS level, inhibiting mitochondrial dysfunction, and NF-κB-mediated Nucling expression. 

Lycopene concentration used in the present study is close to plasma concentration of humans [26,45]. Medin et al. [45] validated the estimated intakes of carotenoid-rich foods from a web-based food recall using carotenoids in blood as an objective reference method in Norwegian study. Mean plasma concentration of lycopene was 0.78 μM of 261 participants in the age groups 8–9 and 12–14 years. Lycopene is the most abundant carotenoid, (approx. 50% of all the 14 different carotenoids) found in the human serum. It has been found at a concentration of approximately 0.5 μM in the human serum [26]. This level fits with concentration of lycopene (0.2 and 0.5 μM) treated in the present study. 

## 5. Conclusions

This study supports the anti-apoptotic effect of lycopene and its underlying molecular mechanism in amyloid-β-stimulated human neuronal SH-SY5Y cells. Lycopene inhibits apoptosis by reducing intracellular and mitochondrial ROS, and by inhibiting mitochondrial damage and NF-κB-related Nucling gene expression in amyloid-β-stimulated SH-SY5Y cells. There is no treatment that can reverse the progression of Alzheimer’s disease. Therefore, lycopene has the potential to be developed as a nutrient supplement for the prevention of AD clinically.

## References

[1] Agostinho P., Cunha R.A., Oliveira C. (2010). Neuroinflammation, oxidative stress and the pathogenesis of Alzheimer’s disease. Curr. Pharm. Des..

[2] Alzheimer’s Association (2013). 2013 Alzheimer’s disease facts and figures. Alzheimers Dement..

[3] Bagyinszky E., Youn Y.C., An S.S., Kim S. (2016). Mutations, associated with early-onset Alzheimer’s disease, discovered in Asian countries. Clin. Interv. Aging.

[4] Liu C.Y., Ohki Y., Tomita T., Osawa S., Reed B.R., Jagust W., Van Berlo V., Jin L.W., Chui H.C., Coppola G. (2017). Two novel mutations in the first tansmembrane domain of Presenilin1 cause young-onset Alzheimer’s disease. J. Alzheimers Dis..

[5] Bao F., Wicklund L., Klein W.L., Nordberg A., Marutle A. (2012). Different amyloid-β oligomer assemblies in Alzheimer brains correlate with age of disease onset and impaired cholinergic activity. Neurobiol. Aging.

[6] Esparza T.J., Zhao H., Cirrito J.R., Cirrito J.R., Cairns N.J., Bateman R.J., Holtzman D.M., Brody D.L. (2013). Amyloidbeta oligomerization in Alzheimer dementia versus high-pathology controls. Ann. Neurol..

[7] Sadigh-Eteghad S., Sabermarouf B., Majdi A., Talebi M., Farhoudi M., Mahmoudi J. (2015). Amyloid-beta: A crucial factor in Alzheimer’s disease. Med. Princ. Pract..

[8] Piemontese L. (2017). New approaches for prevention and treatment of Alzheimer’s disease: A fascinating challenge. Neural Regen. Res..

[9] Libro R., Giacoppo S., Soundara Rajan T., Bramanti P., Mazzon E. (2016). Natural phytochemicals in the treatment and prevention of dementia: An overview. Molecules.

[10] Otaegui-Arrazola A., Amiano P., Elbusto A., Urdaneta E., Martínez-Lage P. (2014). Diet, cognition, and Alzheimer’s disease: Food for thought. Eur. J. Nutr..

[11] Piemontese L. (2017). Plant food supplements with antioxidant properties for the treatment of chronic and neurodegenerative diseases: Benefits or risks?. J. Diet. Suppl..

[12] Fiorini A., Sultana R., Barone E., Cenini G., Perluigi M., Mancuso C., Cai J., Klein J.B., St Clair D., Butterfield D.A. (2012). Lack of p53 affects the expression of several brain mitochondrial proteins: Insights from proteomics into important pathways regulated by p53. PLoS ONE.

[13] Gilgun-Sherki Y., Melamed E., Offen D. (2001). Oxidative stress induced-neurodegenerative diseases: The need for antioxidants that penetrate the blood brain barrier. Neuropharmacology.

[14] Reeve A.K., Krishnan K.J., Turnbull D. (2008). Mitochondrial DNA mutations in disease, aging, and neurodegeneration. Ann. N. Y. Acad. Sci..

[15] Mattson M.P., Gleichmann M., Cheng A. (2008). Mitochondria in neuroplasticity and neurological disorders. Neuron.

[16] Aronis A., Melendez J.A., Golan O., Shilo S., Dicter N., Tirosh O. (2003). Potentiation of Fas-mediated apoptosis by attenuated production of mitochondria-derived reactive oxygen species. Cell Death Differ..

[17] Hashimoto M., Rockenstein E., Crews L., Masliah E. (2003). Role of protein aggregation in mitochondrial dysfunction and neurodegeneration in Alzheimer’s and Parkinson’s diseases. Neuromol. Med..

[18] Karin M., Cao Y., Greten F.R., Li Z.W. (2002). NF-κB in cancer: From innocent bystander to major culprit. Nat. Rev. Cancer.

[19] Radhakrishnan S.K., Kamalakaran S. (2006). Pro-apoptotic role of NF-κB: Implications for cancer therapy. Biochim. Biophys. Acta.

[20] Sakai T., Liu L., Teng X., Mukai-Sakai R., Shimada H., Kaji R., Mitani T., Matsumoto M., Toida K., Ishimura K. (2004). Nucling recruits Apaf-1/pro-caspase-9 complex for the induction of stress-induced apoptosis. J. Biol. Chem..

[21] Tran N.H., Sakai T., Kim S.M., Fukui K. (2010). NF-κB regulates the expression of Nucling, a novel apoptosis regulator, with involvement of proteasome and caspase for its degradation. J. Biochem..

[22] Willcox J.K., Catignani G.L., Lazarus S. (2003). Tomatoes and cardiovascular health. Crit. Rev. Food Sci..

[23] Di Mascio P., Kaiser S., Sies H. (1989). Lycopene as the most efficient biological carotenoid singlet oxygen quencher. Arch. Biochem. Biophys..

[24] Stahl W., Sies H. (1996). Perspectives in biochemistry and biophysics, lycopene: A biologically important carotenoid in humans?. Arch. Biochem. Biophys..

[25] Mayne S.T. (1996). Beta-carotene, carotenoids, and disease prevention in humans. FASEB J..

[26] Gerster H. (1997). The potential role of lycopene for human health. J. Am. Coll. Nutr..

[27] Rao A.V., Agarwal S. (2000). Role of antioxidant lycopene in cancer and heart disease. J. Am. Coll. Nutr..

[28] Chen J., Song Y., Zhang L. (2013). Effect of lycopene supplementation on oxidative stress: An exploratory systematic review and meta-analysis of randomized controlled trials. J. Med. Food.

[29] Gartner C., Stahl W., Sies H. (1997). Lycopene is more bioavailable from tomato paste than from fresh tomatoes. Am. J. Clin. Nutr..

[30] Srivastava S., Srivastava A.K. (2015). Lycopene; chemistry, biosynthesis, metabolism and degradation under various abiotic parameters. J. Food Sci. Technol..

[31] Wu A., Liu R., Dai W., Jie Y., Yu G., Fan X., Huang Q. (2015). Lycopene attenuates early brain injury and inflammation following subarachnoid hemorrhage in rats. Int. J. Clin. Exp. Med..

[32] Khachik F., Carvalho L., Bernstein P.S., Muir G.J., Zhao D.Y., Katz N.B. (2002). Chemistry, distribution, and metabolism of tomato carotenoids and their impact on human health. Exp. Biol. Med..

[33] Hsiao G., Fong T.H., Tzu N.H., Lin K.H., Chou D.S., Sheu J.R. (2004). A potent antioxidant, lycopene, affords neuroprotection against microglia activation and focal cerebral ischemia in rats. In Vivo.

[34] Qu M., Li L., Chen C., Li M., Pei L., Chu F., Yang J., Yu Z., Wang D., Zhou Z. (2011). Protective effects of lycopene against amyloid β-induced neurotoxicity in cultured rat cortical neurons. Neurosci. Lett..

[35] Kumar P., Kalonia H., Kumar A. (2009). Lycopene modulates nitric oxide pathways against 3-nitropropionic acid-induced neurotoxicity. Life Sci..

[36] Pema A., Janakiraman U., Manivasagam T., Thenmozhi A.J. (2015). Neuroprotective effect of lycopene against MPTP induced experimental Parkinson’s disease in mice. Neurosci. Lett..

[37] Nunomura A., Castellani R.J., Zhu X., Moreira P.I., Perry G., Smith M.A. (2006). Involvement of oxidative stress in Alzheimer disease. J. Neuropathol. Exp. Neurol..

[38] Palozza P., Parrone N., Simone R.E., Catalano A. (2010). Lycopene in atherosclerosis prevention: An integrated scheme of the potential mechanisms of action from cell culture studies. Arch. Biochem. Biophys..

[39] Zhang Y., Jiao G., Song C., Gu S., Brown R.E., Zhang J., Zhang P., Gagnon J., Locke S., Stefanova R. (2017). An extract from shrimp processing by-products protects SH-SY5Y cells from neurotoxicity induced by Aβ25–35. Mar. Drugs.

[40] Reed J.C. (2000). Mechanisms of apoptosis. Am. J. Pathol..

[41] Chan P.H. (2004). Mitochondria and neuronal death/survival signaling pathways in cerebral ischemia. Neurochem. Res..

[42] Starkov A.A. (2008). The role of mitochondria in reactive oxygen species metabolism and signaling. Ann. N. Y. Acad. Sci..

[43] Maes M., Fišar Z., Medina M., Scapagnini G., Nowak G., Berk M. (2012). New drug targets in depression: Inflammatory, cell-mediated immune, oxidative and nitrosative stress, mitochondrial, antioxidant, and neuroprogressive pathways. And new drug candidates—Nrf2 activators and GSK-3 inhibitors. Inflammopharmacology.

[44] Lim S., Hwang S.W., Yu J.H., Lim J.W., Kim H. (2017). Lycopene inhibits regulator of calcineurin 1-mediated apoptosis by reducing oxidative stress and down-regulating Nucling in neuronal cells. Mol. Nutr. Food Res..

[45] Medin A.C., Carlsen M.H., Andersen L.F. (2016). Associations between reported intakes of carotenoid-rich foods and concentrations of carotenoids in plasma: A validation study of a web-based food recall for children and adolescents. Public Health Nutr..

